# Transcription-coupled changes to chromatin underpin gene silencing by transcriptional interference

**DOI:** 10.1093/nar/gkw801

**Published:** 2016-09-08

**Authors:** Ryan Ard, Robin C. Allshire

**Affiliations:** Wellcome Trust Centre for Cell Biology and Institute of Cell Biology, School of Biological Sciences, The University of Edinburgh, Edinburgh EH9 3BF, Scotland, UK

## Abstract

Long non-coding RNA (lncRNA) transcription into a downstream promoter frequently results in transcriptional interference. However, the mechanism of this repression is not fully understood. We recently showed that drug tolerance in fission yeast *Schizosaccharomyces pombe* is controlled by lncRNA transcription upstream of the *tgp1^+^* permease gene. Here we demonstrate that transcriptional interference of *tgp1^+^* involves several transcription-coupled chromatin changes mediated by conserved elongation factors Set2, Clr6CII, Spt6 and FACT. These factors are known to travel with RNAPII and establish repressive chromatin in order to limit aberrant transcription initiation from cryptic promoters present in gene bodies. We therefore conclude that conserved RNAPII-associated mechanisms exist to both suppress intragenic cryptic promoters during genic transcription and to repress gene promoters by transcriptional interference. Our analyses also demonstrate that key mechanistic features of transcriptional interference are shared between *S. pombe* and the highly divergent budding yeast *Saccharomyces cerevisiae*. Thus, transcriptional interference is an ancient, conserved mechanism for tightly controlling gene expression. Our mechanistic insights allowed us to predict and validate a second example of transcriptional interference involving the *S. pombe pho1^+^* gene. Given that eukaryotic genomes are pervasively transcribed, transcriptional interference likely represents a more general feature of gene regulation than is currently appreciated.

## INTRODUCTION

The organization of DNA into chromatin poses a significant physical challenge to eukaryotic transcription. In order to activate gene expression, chromatin must be opened in manner that allows transcription factors, co-activators, and RNA polymerase II (RNAPII) access to the DNA template (1). Active eukaryotic promoters therefore exhibit nucleosome-depleted regions immediately upstream of the transcription start site (TSS) (2). In contrast, developmentally regulated and stress-response gene promoters frequently display increased nucleosome density, which limits gene expression by masking key regulatory sequences in DNA when repression of such genes is required.

Numerous chromatin remodelers, histone chaperones, and chromatin-modifying activities cooperate to reorganize nucleosomes and permit transcription into gene bodies (3). These factors are often recruited directly or indirectly by histone modifications on nearby nucleosomes and/or by specific post-translational modifications to the C-terminal domain (CTD) of Rpb1, the largest subunit of RNAPII (4). For example, Ser-5 phosphorylation (Ser5P) on the Rpb1 CTD indirectly recruits histone methyltransferase (HMT) activity, which transfers three methyl groups on to lysine 4 of histone H3 (H3K4me3) at active promoters (5). This active histone mark is thought to simultaneously prevent the binding of repressive complexes and recruit histone acetyl transferases (HATs), which transfer acetyl groups to lysine residues on histone tails to bring about a more open chromatin structure that is permissive to transcription initiation (6–8). In contrast, histone deacetylase complexes (HDACs) remove acetyl groups from histone tails, while other HMTs deposit repressive histone marks (e.g. H3K9me2/3). Such activities create a much less accessible chromatin environment that silences gene expression, termed heterochromatin (9).

As RNAPII travels away from the initiation site, the Rpb1 CTD gradually loses Ser5P and acquires Ser2P (10,11). The Ser2P form of the Rpb1 CTD recruits the HMT Set2, which methylates H3K36 on nucleosomes positioned over the body of transcribed genes (12,13). The H3K36me3 mark serves as a docking site for Rpd3S (14), an HDAC that is co-transcriptionally recruited by direct interactions with RNAPII (15,16). Importantly, Rpd3S establishes a hypoacetylated chromatin environment over gene bodies to prevent aberrant transcription initiation from cryptic intragenic promoters (17,18). Thus, crosstalk between RNAPII and the underlying chromatin tightly control chromatin states and gene expression.

Over the last decade, the location of complexes such as RNAPII and nucleosomes, including their variant modified forms, have been mapped genome wide in various organisms and cell types (19). Such studies complemented transcript-profiling analyses and revealed that eukaryotic genomes are pervasively transcribed by RNAPII (20). Apart from just generating messenger RNAs (mRNAs) from protein-coding genes, we now know that RNAPII also synthesizes long non-coding RNAs (lncRNAs) that overlap protein-coding genes on the sense and antisense strands, from within introns, and from intergenic regions (21). Although mRNAs and lncRNAs are both produced by RNAPII, these transcripts have notably different fates. Unlike stable mature mRNAs that are exported to the cytoplasm for protein synthesis, lncRNAs remain predominantly nuclear and are often rapidly degraded by RNA decay pathways (22). In addition, most lncRNAs are poorly conserved in primary nucleotide sequence when compared with mRNAs (23). While low steady-state levels and poor sequence conservation are obvious challenges for characterizing lncRNA functions, substantial progress has been made into assigning biological functions to an accumulating number of individual lncRNAs (24). However, this difficult task is confounded by the fact that subsequent studies regularly overturn the interpretations of previous reports (25,26), making lncRNAs arguably the least understood and most contentious products of eukaryotic genomes.

An emerging paradigm is that many lncRNAs contribute to the regulation of gene expression. For example, several individual lncRNAs have been reported to interact with and/or direct chromatin-modifiers to control gene expression, while others have been proposed to recruit transcriptional activators, repressors, or components of the transcription machinery itself (27). Although there is evidence that some lncRNAs regulate distant genes in *trans*, lncRNAs more frequently influence adjacent gene expression in *cis*. Notably, antisense transcription can compete with transcription on the sense strand to regulate gene expression (28). Functionally, this balance of sense and antisense transcription is required to control the expression of numerous meiotic and stress-response genes in different yeast species (29,30). The act of intergenic lncRNA transcription can also have a profound impact on the expression of nearby genes. For example, the activation of the *fbp1^+^* gene in fission yeast (*Schizosaccharomyces pombe*) occurs in response to glucose starvation and requires upstream lncRNA transcription to evict promoter-associated repressors and permit transcription factor binding (31,32). Conversely, a phenomenon known as ‘transcriptional interference’ often involves transcription of lncRNAs into downstream gene promoters to repress their expression. While this form of gene repression has been documented in many organisms, ranging from prokaryotes to higher eukaryotes (33–39), a comprehensive molecular understanding of the mechanism is lacking. Perhaps the best-studied eukaryotic example of transcriptional interference involves lncRNA transcription into the budding yeast *S. cerevisiae SER3* gene promoter. In serine-rich growth conditions, the act of lncRNA transcription prohibits transcription factor access to repress *SER3* expression (36). Additional examples of gene regulation by the act of lncRNA transcription, rather than by the RNAs produced (40), underscore the biological significance of transcribing lncRNAs, even if the RNA products are themselves not functional.

Recently we reported that the *S. pombe* permease gene *tgp1^+^* is regulated by transcriptional interference in response to changes in extracellular phosphate levels (34). While lncRNA transcription into the *tgp1^+^* promoter is associated with increased nucleosome density and *tgp1^+^* repression does not require the RNA interference (RNAi) pathway or heterochromatin components, little is known about the mechanism that mediates this repression. Here we show that numerous conserved transcriptional elongation factors (including Set2, the Rpd3S homolog Clr6CII, and histone chaperones Spt6 and FACT) are required to establish a repressive chromatin environment over the *tgp1^+^* promoter in the wake of upstream initiating lncRNA transcription. Furthermore, the identification of key factors associated with transcriptional interference at the *tgp1^+^* locus allowed us to demonstrate that a second phosphate-response gene (*pho1^+^*) is regulated by a similar mechanism. We conclude that the mechanism of transcriptional interference appears to be well conserved between *S. pombe*, which retains active RNAi, and the distantly related budding yeast *S. cerevisiae*, which lacks the RNAi pathway. Thus, RNAi does not play a role in the mechanisms that repress genes by transcriptional interference. Rather, upstream lncRNA transcription-coupled changes in promoter chromatin status underpin gene silencing by transcriptional interference in two evolutionarily distant eukaryotes.

## MATERIALS AND METHODS

### Yeast strains, plasmids, and standard techniques

*S. pombe* strains used in this study are listed in Supplementary Table S1. Standard methods were used for fission yeast growth, genetics and manipulations (41). All strains were grown in YES medium (Yeast extract plus supplements) at 32°C, unless otherwise indicated. For phosphate starvation experiments, cells were grown to mid-log phase in EMMS (Edinburgh minimum medium with supplements) (Formedium), washed twice in dH_2_O, and then grown for indicated times in EMMS without phosphates (Formedium). *nmt1-nc-tgp1* cells were grown in phosphate-rich PMG (Pombe minimal glutamate) medium in the presence or absence of thiamine (15 μM). Endogenous genetic manipulations were carried out by lithium acetate transformation. Selections were performed on PMG/agar plates with according auxotrophy or on YES/agar plates with appropriate antibiotic(s) and grown at 32°C. Colony PCR confirmed all endogenous genetic modifications and crosses. For spotting assays, serial (1:4) dilutions of equal numbers of cells were spotted onto YES/agar and grown at 32°C. For drug-sensitivity experiments, cells were spotted onto YES/agar with vehicle (DMSO) or TBZ (20 μg/ml), HU (10 mM), caffeine (15 mM).

### Chromatin immunoprecipitation

Cells were grown to mid-log phase at 32°C. For phosphate starvation experiments, cells in mid-log phase were washed twice in dH_2_O before being grown in EMMS without phosphates (-PO_4_) for 6 h. For temperature sensitive strains, cells were shifted from 32°C to the restrictive temperature of 36°C for 1 h. ChIP was performed essentially as described (42). Briefly, cells were fixed with 1% paraformaldehyde (PFA) for 15 min at room temperature. Cells were lysed by bead beating (Biospec Products) and sonicated using a Bioruptor (Diagenode) sonicator at 5°C on high for a total of 30 min (30 s ON/OFF cycles). 2 μl Ser2P Rpb1 CTD antibody (ab5095; Abcam), 2 μl H3 antibody (ab1791; Abcam), 2 μl H3K9ac antibody (39137; Active Motif), 2 μl H4K12ac antibody (39165; Active Motif), 2 μl H3K36me3 antibody (61101; Active Motif), 1 μl H3K9me2 antibody (5.1.1), and 2 μl GFP antibody (A11122; Life Technologies) were used per ChIP. Quantitative analysis was performed by qPCR.

### RNA analysis

RNA was isolated from *S. pombe* using RNeasy Mini- or Midi-Kits as per manufacturer's instructions (Qiagen). For quantitative reverse transcriptase PCR (RT-qPCR) experiments, first strand cDNA synthesis was performed on Turbo DNase (Life Technologies) treated RNA using random hexamers and Superscript IV (Invitrogen) as per manufacturer's instructions. Negative controls lacking the reverse transcriptase enzyme (-RT) were performed alongside all RT-qPCR experiments. Quantitative analysis was performed by qPCR. To make RNA probes for northern analysis, DNA fragments specific to target transcripts were amplified from genomic DNA by PCR and gel-purified using the Wizard^®^ SV Gel and PCR Clean-Up System (Promega). The T7 promoter was equipped at the end of the DNA fragment using an oligonucleotide containing T7 promoter sequence at the 5′-end (TAATACGACTCACTATAGGGAGA). The T7 promoter containing PCR products were transcribed *in vitro* using the MaxiScript T7 Kit (Ambion) to produce UTP-(α^32^P)-labelled RNA probes following the manufacturer's instructions. Unincorporated radionucleotides were removed using NucAway Spin columns (Life Technologies) according to manufacturer's instructions. The UTP-(α^32^P)-labelled RNA probes were hybridized to membranes overnight in church buffer (0.5 M Na_2_HPO_4_ pH 7.2, 1 mM EDTA, 7% SDS) at 68°C in a rotating oven. Hybridized membranes were washed twice in a pre-warmed buffer containing 2× SSC and 0.1% SDS for 30 min at 68°C followed by two washes in a buffer containing 0.5× SSC and 0.1% SDS for 15 min at 68°C. To detect transcripts, northern blots were analyzed after 1–2 days of exposure on a Phosphor Screen (Molecular Dynamics) using a Typhoon Phosphorimager (GE Healthcare Life Sciences).

### qPCR

Primers used in this study are listed in Supplementary Table S2. Quantitative real-time PCR (qPCR) was performed using SYBR Green on a Roche Lightcycler. Data was analyzed with LightCycler 480 Software 1.5.0.39. RT-qPCR levels were calculated by normalizing product of interest to an internal reference gene (*act1*^+^). Expression levels were expressed relative to levels detected in wild-type cells. ChIP enrichments were calculated as the ratio of product of interest from IP sample normalized to the corresponding input sample and expressed as ‘%IP.’ Error bars represent standard deviation resulting from at least three independent replicates.

## RESULTS

### *nc-tgp1* transcription establishes Set2-dependent H3K36me3 over the *tgp1^+^* promoter

**Figure 1.**
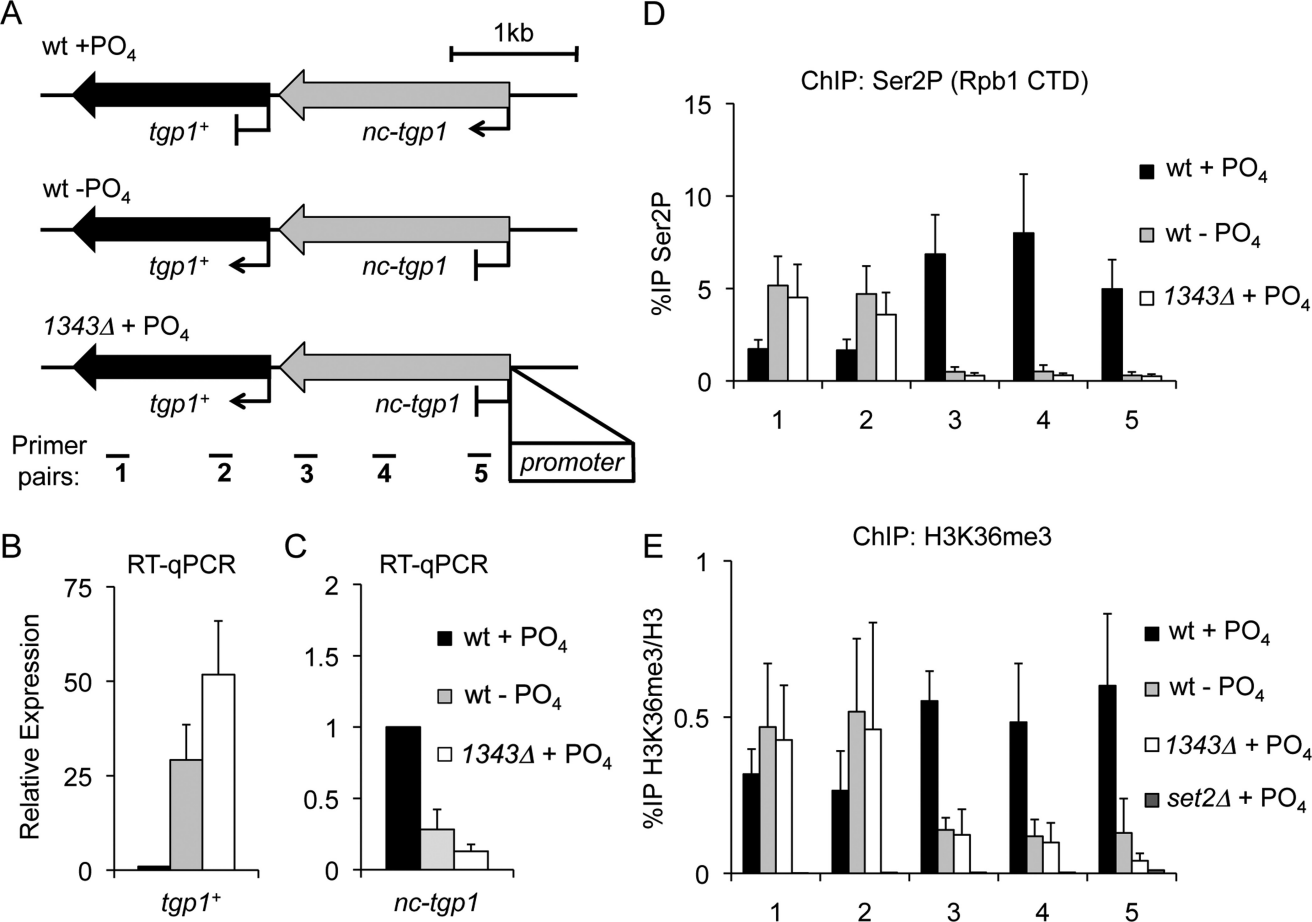
*nc-tgp1* transcription increases H3K36me3 levels over the *tgp1^+^* promoter. (**A**) Schematic representation of the *tgp1^+^* locus, including the *nc-tgp1* lncRNA gene located immediately upstream. In phosphate-rich conditions (+PO_4_) the *tgp1^+^* gene is repressed by *nc-tgp1* transcription into the *tgp1^+^* promoter. In contrast, reduced *nc-tgp1* transcription, in response to phosphate starvation (−PO_4_) or following deletion of the *nc-tgp1* promoter (*1343Δ*), permits *tgp1^+^* induction (34). Primer pairs spaced over the *tgp1^+^* locus are displayed below. (**B** and **C**) RT-qPCR analysis of *tgp1^+^* mRNA levels (primer pair 1) and upstream *nc-tgp1* lncRNA levels (primer pair 5) in wild-type cells grown in the presence or absence of phosphate, as well as in phosphate-replete*1343Δ* cells. (**D**) ChIP-qPCR analysis of the Ser2P form of elongating RNAPII over the *tgp1^+^* locus in response to changes in phosphate availability/*nc-tgp1* transcription. (**E**) ChIP-qPCR analysis of H3K36me3 over the *tgp1^+^* locus in response to changes in phosphate availability/*nc-tgp1* transcription. Error bars represent standard deviation resulting from at least three independent experiments.

Maintaining stable cellular phosphate levels is a challenge since inorganic phosphate availability can fluctuate unpredictably. To combat this, organisms have evolved complex strategies to sense extracellular phosphate levels and integrate this information into a transcriptional response that increases survival. The glycerophosphodiester permease gene *tgp1^+^* is a core component of the phosphate regulon in *S. pombe* and is activated in phosphate-limited conditions by the Pho7 transcription factor (43). In addition, Pho7 has been shown to bind directly to phosphate regulated gene promoters, such as *tgp1^+^* and *pho1^+^* promoters (43). When external phosphate levels are high, an upstream exosome-sensitive lncRNA termed *nc-tgp1* is transcribed in tandem with the *tgp1^+^* gene and corresponds with increased nucleosome density and decreased Pho7 binding over the *tgp1^+^* promoter (Figure 1A) (34,44,45). Although this repression occurs in an RNAi and heterochromatin-independent manner, it is possible that other transcription-coupled changes in chromatin status contribute to transcriptional interference at the *tgp1^+^* locus.

To assess the distribution of the elongating form of RNAPII over the *tgp1^+^* locus in response to phosphate availability, the levels of Ser2 phosphorylated RNAPII (Rpb1-Ser2P) were assessed by quantitative ChIP in wild-type cells grown in the presence or absence of phosphate. These analyses revealed the presence of increased levels of Rpb1-Ser2P over the *tgp1^+^* promoter in phosphate-replete cells (i.e. when the repressive lncRNA *nc-tgp1* is actively transcribed), compared to phosphate-starved cells (Figure 1B–D). This finding is consistent with the fact that following phosphate starvation *tgp1^+^* induction is accompanied by reduced *nc-tgp1* transcription (34).

**Figure 2.**
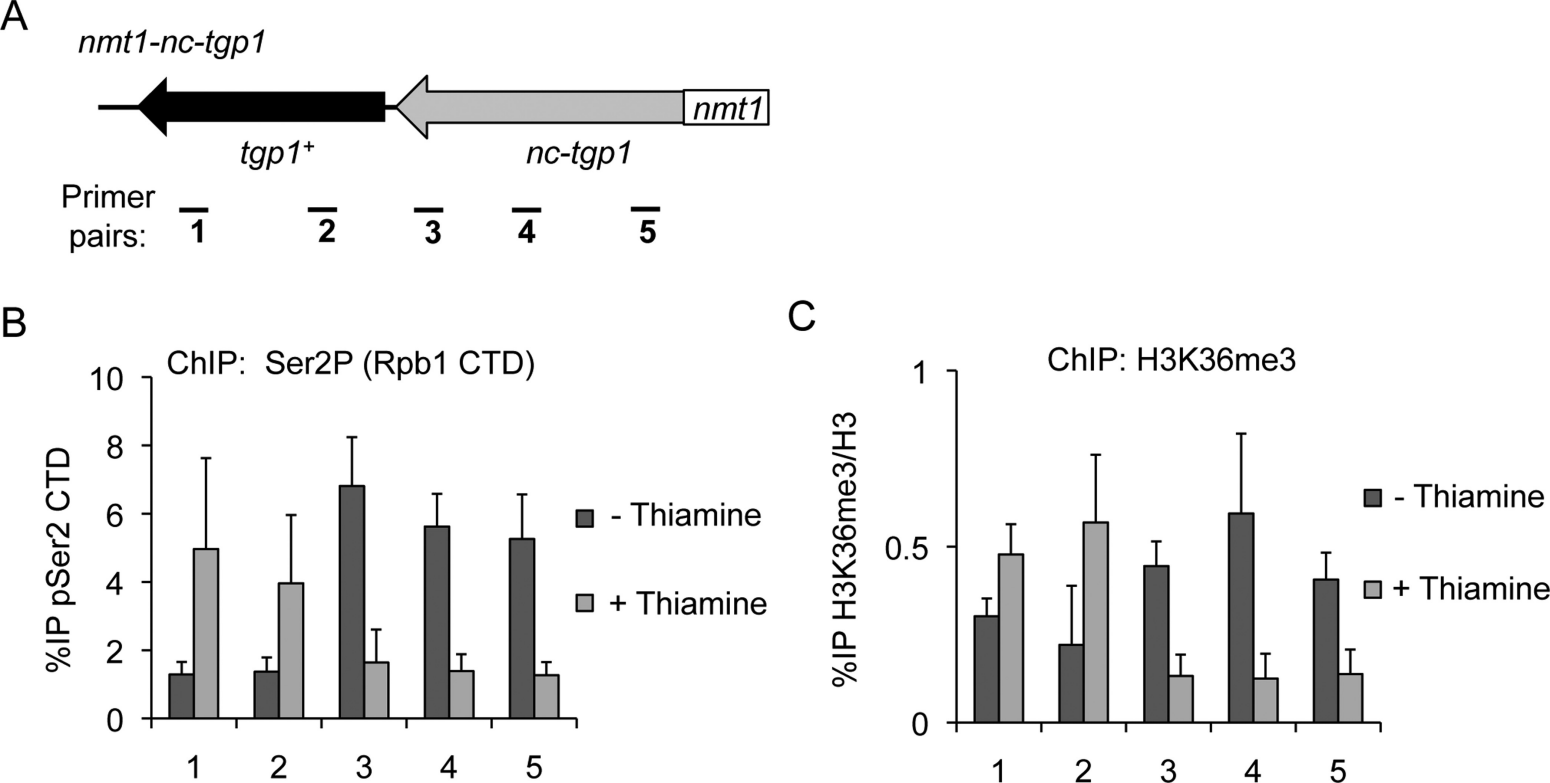
*nmt1*-driven *nc-tgp1* transcription directs the accumulation of H3K36me3 over the *tgp1^+^* promoter in response to thiamine. (**A**) Replacing the *nc-tgp1* promoter with a strong thiamine-regulated *nmt1* promoter brings *nc-tgp1* transcription under the control of thiamine. (**B**) ChIP-qPCR for Ser2P, the elongating form of RNAPII, over the *tgp1+* locus in *nmt1-nc-tgp1* cells grown in the presence or absence of thiamine. (**C**) ChIP-qPCR for H3K36me3 levels over the *tgp1+* locus in *nmt1-nc-tgp1* cells grown in the presence or absence of thiamine. Error bars represent standard deviation resulting from at least three independent replicates.

It is well established that the elongating Ser2P form of RNAPII recruits the H3K36 HMT Set2 (12,13). Examination of available ChIP-seq data revealed enriched levels of Set2 at the *tgp1^+^* promoter in repressed phosphate-rich conditions (Supplementary Figure S1) (46), suggesting Set2 might contribute to *tgp1^+^* regulation. Indeed, active *nc-tgp1* transcription in phosphate-replete cells correlated with increased levels of H3K36me3 over the *tgp1^+^* promoter region (Figure 1E). To determine if *nc-tgp1* transcription into the *tgp1^+^* promoter is required to establish Set2-dependent H3K36me3, we performed these analyses in cells lacking the promoter that drives *nc-tgp1* transcription (*1343Δ*). This manipulation prevented *nc-tgp1* transcription in phosphate-rich conditions and results in constitutive *tgp1^+^* expression (Figure 1D). Consistent with H3K36me3 being deposited during *nc-tgp1* transcription elongation, removal of the *nc-tgp1* promoter resulted in significantly reduced H3K36me3 levels upstream of the *tgp1^+^* gene in the presence of phosphate (Figure 1E). Additionally, replacement of the *nc-tgp1* promoter with a strong thiamine-repressive promoter (*nmt1*) brings *nc-tgp1* under the control of thiamine, rather than phosphate. In these cells, Rpb1-Ser2P and H3K36me3 levels were significantly higher in the absence of thiamine (i.e. when *nmt1-nc-tgp1* is transcribed) than in cells grown in the presence of thiamine (i.e. when *nmt1-nc-tgp1* transcription is reduced) (Figure 2). This manipulation demonstrates that *nc-tgp1* transcription from even a heterologous promoter is sufficient to cause H3K36me3 to accumulate over the *tgp1^+^* promoter. Together, these results demonstrate that the act of *nc-tgp1* transcription stimulates Set2-dependent deposition of H3K36me3 over the *tgp1^+^* promoter.

### Loss of H3K36 methylation induces *tgp1*^+^ expression

**Figure 3.**
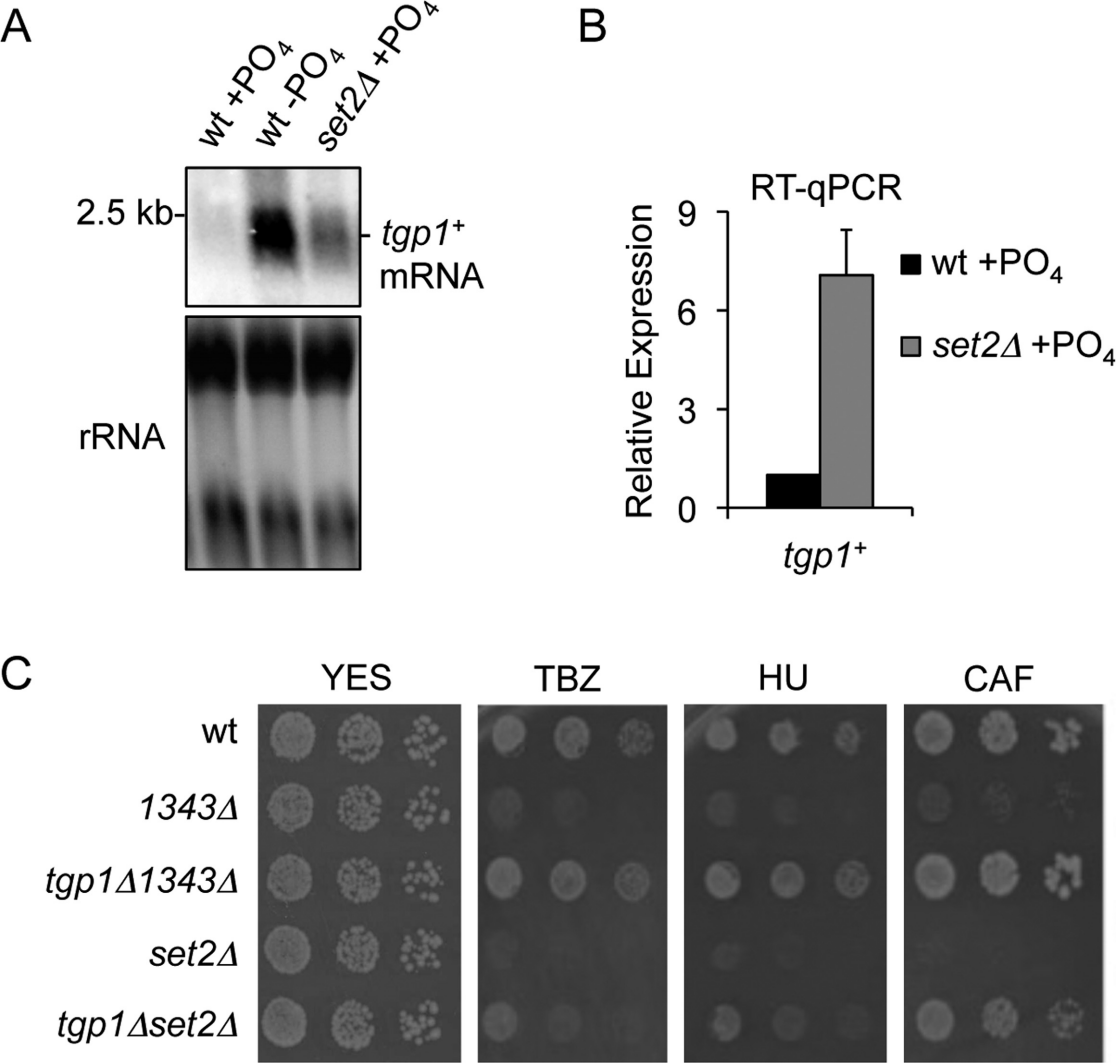
Set2 represses *tgp1^+^* expression. (**A**) Northern analysis of *tgp1^+^* mRNA levels in wild-type cells grown in the presence or absence of phosphate and *set2Δ* cells grown in the presence of phosphate. (**B**) RT-qPCR analysis of *tgp1^+^* mRNA levels in phosphate-replete wild-type and *set2Δ* cells. Error bars represent standard deviation resulting from three independent experiments. (**C**) Serial dilutions of wild-type cells, *1343Δ* (*nc-tgp1* promoter deleted; constitutive *tgp1^+^* expression), the *tgp1Δ1343Δ* double mutant, *set2Δ*, and the *tgp1Δset2Δ* double mutant spotted on non-selective YES medium or in the presence of the following compounds: thiabendazole (TBZ: 20 μg/mL), hydroxyurea (HU: 10 mM), or caffeine (CAF: 15 mM).

Having established that the act of transcribing *nc-tgp1* into the *tgp1^+^* promoter directs the accumulation of H3K36me3, we next sought to determine if the presence of this mark plays a direct role in curbing *tgp1^+^* expression. We measured *tgp1^+^* mRNA levels in wild-type cells grown in the presence or absence of phosphate as well as in phosphate-replete cells lacking H3K36 methylation (i.e. *set2Δ* cells). As expected, northern analysis detected the *tgp1^+^* mRNA in phosphate-starved cells but not cells grown in the presence of phosphate (repressed condition) (Figure 3A). Notably, elevated levels of the *tgp1^+^* mRNA were detected in *set2Δ* cells grown in repressive phosphate-rich media (Figure 3A). Quantitative analyses confirmed a ∼7-fold increase in *tgp1^+^* expression in cells lacking Set2 (Figure 3B). This finding confirms microarray data indicating that *tgp1^+^* expression increases in *set2Δ* cells (48). While this increase in *tgp1^+^* mRNA levels is indeed significant, it is substantially lower than that detected in phosphate-starved wild-type cells (Figure 3A), or phosphate-replete *1343Δ* cells (Supplementary Figure S1), suggesting that other factors must cooperate with Set2 to mediate full repression of *tgp1^+^*. Nonetheless, these results suggest that Set2 activity contributes to the efficient silencing of *tgp1^+^* mediated by upstream *nc-tgp1* transcription.

Constitutive *tgp1^+^* expression renders *S. pombe* cells hypersensitive to growth in the presence of various compounds (34), such as the microtubule destabilizing drug thiabendazole (TBZ), DNA-synthesis inhibitor hydroxyurea (HU) and caffeine (CAF), a potent inhibitor of cyclic AMP phosphodiesterase. This *tgp1^+^*-dependent phenotype must result from greater drug uptake in cells expressing elevated levels of the *tgp1^+^* permease. Interestingly, in our growth assays we found that *set2Δ* cells are also sensitive to growth in the presence of these three compounds (Figure 3C). Deletion of the *tgp1^+^* gene in the *set2Δ* background revealed that cells lacking both Tgp1 and Set2 (*tgp1Δset2Δ*) were more resistant to growth in the presence of TBZ, HU, and CAF than cells lacking Set2 alone (Figure 3C). Thus, increased *tgp1^+^*expression contributes to the increased drug sensitivity observed in *set2Δ* cells. Overall, these findings support a role for lncRNA transcription-coupled Set2 recruitment in the repression of *tgp1^+^* by transcriptional interference.

### HDAC Clr6CII participates in lncRNA-dependent repression of *tgp1*^+^

**Figure 4.**
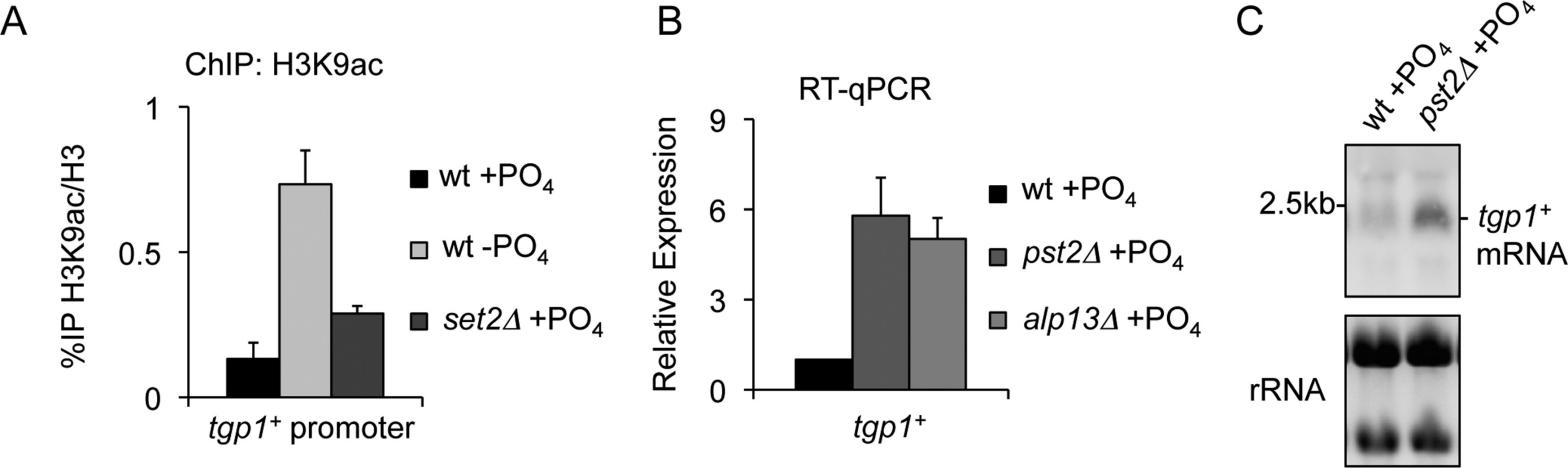
Clr6CII HDAC activity is required to suppress *tgp1^+^* expression. (**A**) The levels of acetyl-histones (H3K9ac) at the *tgp1^+^* promoter primer pair 3 (see Figure 1A) were measure by ChIP-qPCR in wild-type cells grown in the presence or absence of phosphate and *set2Δ* cells grown in the presence of phosphate. (**B**) RT-qPCR analysis of *tgp1^+^* mRNA levels in phosphate-replete wild-type cells and cells lacking either Pst2 or Alp13, each of which are critical HDAC subunits of Clr6CII^Rpd3S^. Error bars represent standard deviation resulting from three independent replicates. (**C**) Northern analysis of *tgp1^+^* mRNA levels in wild-type cells grown in the presence or absence of phosphate and in phosphate-replete cells with compromised Clr6CII^Rpd3S^ activity (*pst2Δ*).

Histone acetylation promotes an open chromatin configuration that accompanies transcriptional activation (48). We predicted that H3K36me3 deposited over the *tgp1^+^* promoter during *nc-tgp1* transcription would recruit the *S. pombe* Rpd3S complex (termed Clr6CII) and that this would result in hypoacetylated chromatin over the region upstream of *tgp1^+^*. ChIP analyses revealed that the levels of histone acetylation (H3K9ac and H4K12ac) over the *tgp1^+^* promoter were highest in cells with active *tgp1^+^* expression (phosphate-starved cells), while much lower levels of histone acetylation were observed in wild-type cells grown in the presence of phosphate (Figure 4A and Supplementary Figure S1). In addition, the partial activation of *tgp1^+^* expression in *set2Δ* cells was also accompanied by increased histone acetylation levels over the *tgp1^+^* promoter (Figure 4A), suggesting histone deacetylation is involved in mediating transcriptional interference at the *tgp1^+^* gene. To directly test if Clr6CII^Rpd3S^ contributes to *tgp1^+^* repression, *tgp1^+^* mRNA levels were measured in cells lacking Pst2 or Alp13, two critical subunits of the HDAC Clr6CII^Rpd3S^. Cells with compromised Clr6CII^Rpd3S^ activity (*pst2Δ* or *alp13Δ*) exhibited partial *tgp1^+^* induction (Figure 4B and 4C), thereby demonstrating that Clr6CII^Rpd3S^ contributes to *tgp1^+^* repression. Support for this conclusion is also provided by previous microarray data which detected increased *tgp1^+^* expression in the *clr6-1* mutant (49). Collectively, our analyses demonstrate that *nc-tgp1* transcription in phosphate-rich conditions directs the accumulation of Set2-dependent H3K36me3 over the *tgp1^+^* promoter; a mark that promotes Clr6CII^Rpd3S^ recruitment and activity to further represses local chromatin by histone deacetylation. Importantly, our results indicate that specific chromatin features cooperate to reinforce *tgp1^+^* silencing by transcriptional interference.

### *tgp1*^+^ repression by transcriptional interference requires histone chaperones Spt6 and FACT

**Figure 5.**
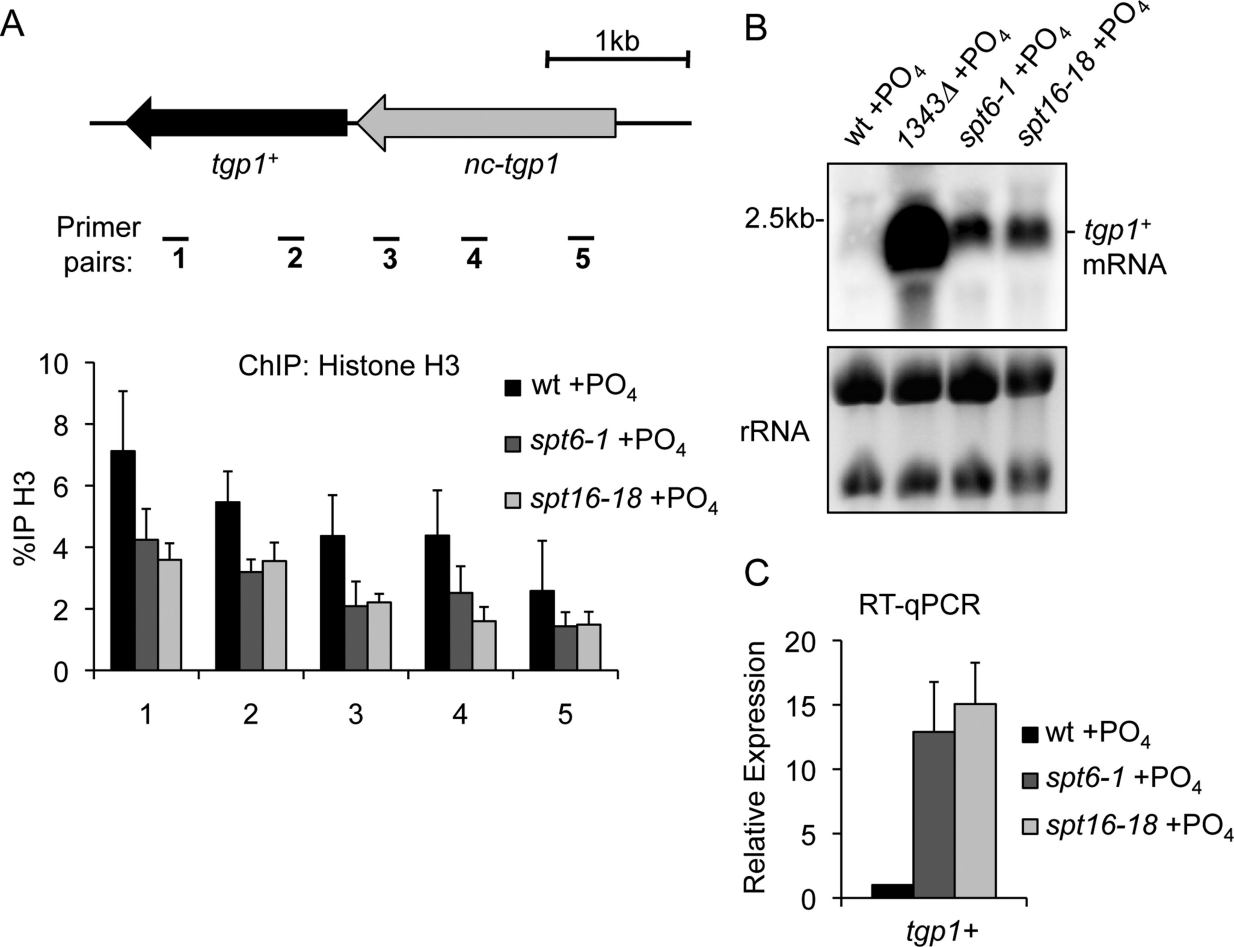
Spt6 and FACT are required to suppress *tgp1^+^* expression. (**A**) Histone density over the *tgp1^+^* locus was measured by histone H3 ChIP-qPCR experiments in phosphate-replete wild-type cells, Stp6 mutant cells (*spt6-1*), and cells lacking the activity of the FACT subunit Spt16 (*spt16-18*) grown for 1 h at the restrictive temperature of 36°C. (**B**) Northern analysis of *tgp1^+^* mRNA levels in wild-type cells, *1343Δ* cells with constitutive *tgp1^+^* expression (i.e. cells lacking the promoter that drives repressive *nc-tgp1* transcription), and in cells with compromised Spt6 or FACT activity grown at the restrictive temperature for 1 h. (**C**) RT-qPCR analysis of *tgp1^+^* mRNA levels in phosphate-replete wild-type cells, *spt6-1* cells, and *spt16-18* cells grown at the restrictive temperature for 1 h. Error bars represent standard deviation resulting from at least three independent replicates.

Active promoters display nucleosome-depleted regions, whereas repressed gene promoters exhibit increased nucleosome density (2). Indeed, increased nucleosome density is detected at the *tgp1^+^* promoter when it is repressed by upstream *nc-tgp1* transcription (34). Histone chaperones Spt6 and FACT (facilitates chromatin transcription) travel with RNAPII during transcription elongation and perform two main tasks: (i) disassembly of nucleosomes to permit transcription elongation and (ii) reassembly of nucleosomes in a manner that helps suppress intragenic transcription initiation (50). Thus, these factors are prime candidates to explain the high nucleosome density over the *tgp1^+^* promoter correlating with *nc-tgp1* transcription. Our ChIP analyses revealed that histone H3 levels in phosphate-replete cells (repressed condition) lacking the activity of Spt6 (*spt6-1*) or FACT (*spt16-18*) are substantially reduced over the *tgp1^+^* promoter (Figure 5A). Decreased histone H3 levels over the *tgp1^+^* locus in *spt6-1* and *spt16-18* mutants also corresponded with accumulating levels of the *tgp1^+^* mRNA (Figure 5B and C). Cells lacking the Pob3 subunit of the FACT complex also displayed increased levels of *tgp1^+^* expression (Supplementary Figure S1). We conclude that the histone chaperones Spt6 and FACT are instrumental in mediating differences in nucleosome density to repress *tgp1^+^* activation in response to *nc-tgp1* transcription. However, we note that similar to results obtained in mutants defective in Set2 and Clr6CII, loss of either Spt6 or FACT leads to only the partial activation of *tgp1^+^* expression. It is likely that these and other elongation factors cooperate to mediate complete transcriptional interference.

### *pho1*^+^ repression is mediated by transcriptional interference

**Figure 6.**
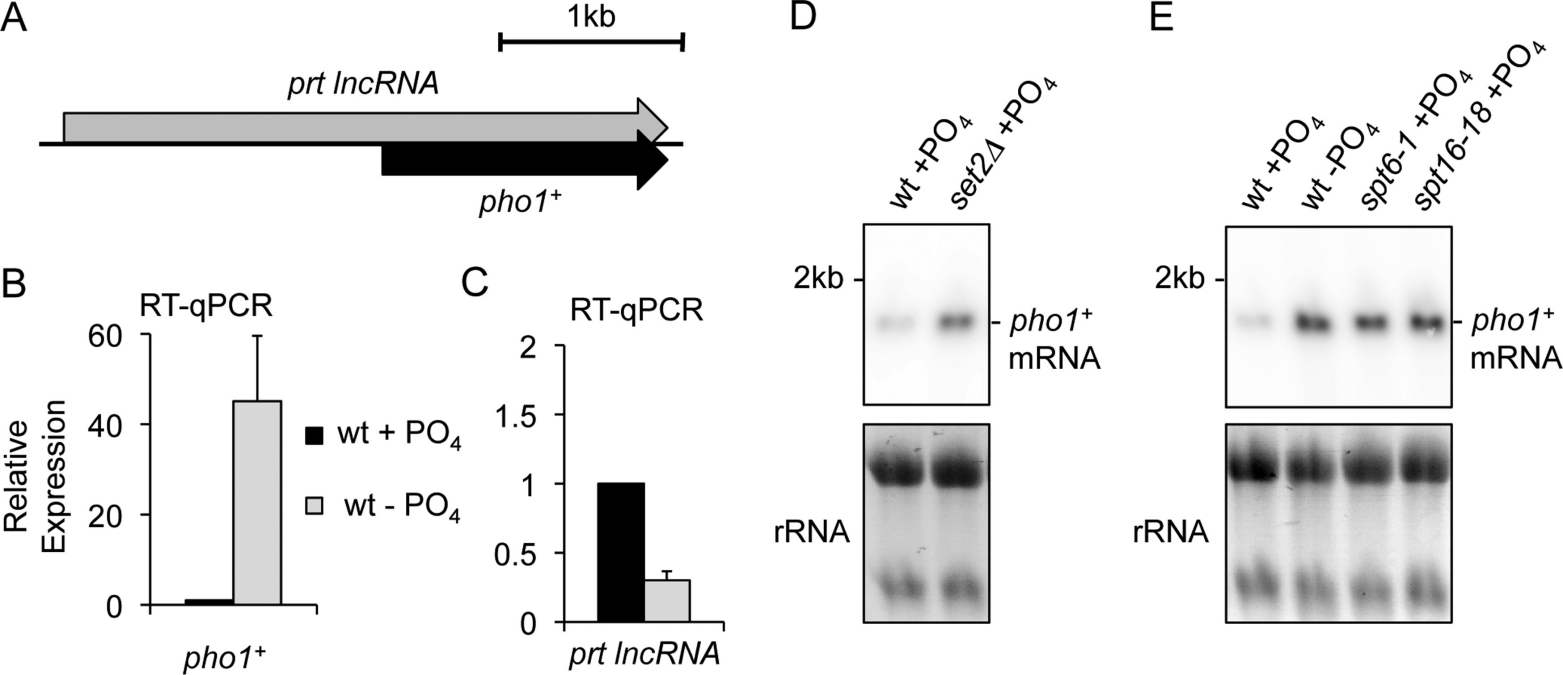
Elongation factors involved in co-transcriptional chromatin reorganization are required to suppress *pho1^+^* expression. (**A**) Schematic depiction of the *pho1^+^* locus, including the lncRNA *prt* gene that occupies the *pho1^+^* promoter and overlaps the gene body downstream. (**B** and **C**) RT-qPCR analysis of *pho1^+^* mRNA levels and upstream *prt* lncRNA levels in wild-type cells grown in the presence or absence of phosphate using primer pairs 1 and 4, respectively (see Figure 7A). Error bars represent standard deviation resulting from at least three independent replicates. (**D** and **E**) Northern analysis of *tgp1^+^* mRNA levels in wild-type cells grown in the presence or absence of phosphate and in phosphate-replete *set2Δ, spt6-1*, and *spt16-18* cells.

The above analyses demonstrate that *tgp1^+^* regulation by upstream lncRNA transcription requires several conserved transcription elongation factors to direct the assembly of repressive chromatin over the *tgp1^+^* promoter. To identify other genes that may be similarly regulated we surveyed other nutritionally regulated genes in *S. pombe* that display similar hallmarks of transcriptional interference (e.g. upstream initiating lncRNA, elevated Set2 levels at promoters, etc.). A prime candidate was the *pho1^+^* gene, which exhibits exosome-sensitive lncRNA transcription that initiates upstream and overlaps the phosphate-regulated *pho1^+^* gene (Figure 6A) (45). Impaired exosome-mediated degradation of this overlapping lncRNA was previously reported to result in the accumulation of H3K9 methylation at the *pho1^+^* gene (45,51). Nevertheless, the mechanism of *pho1^+^* regulation in wild-type cells remain unclear since genome-wide analyses of H3K9 methylation do not detect this repressive heterochromatin mark at the *pho1^+^* gene in wild-type cells grown under normally repressive phosphate-rich conditions (52–54). In contrast, ChIP-seq analysis reveal that Set2 is enriched upstream of the *pho1^+^* gene in phosphate-rich media (Supplementary Figure S2) (46). Therefore, a mechanism of transcriptional interference similar to that observed for *tgp1^+^* regulation might also be involved in silencing *pho1^+^* expression in wild-type cells grown in the presence of phosphate.

Consistent with *pho1^+^* regulation by transcriptional interference, our analyses demonstrate that *pho1^+^* expression was induced in phosphate-replete cells lacking Set2 (Figure 6D). We note that increased *pho1^+^* transcription is also evident in expression profiling data from *set2Δ* cells (47). In addition, *pho1^+^* mRNA levels similarly accumulated in cells lacking the activity of Clr6CII^Rpd3S^ (p*st2Δ* or *alp13Δ*), Spt6 (*spt6-1*) and FACT (*spt16-18* or *pob3Δ*) (Figure 6E, and Supplementary Figure S2). These analyses show that *pho1^+^* is induced in the absence of factors implicated in transcriptional interference (Supplementary Figure S2). Notably, *pho1^+^* is not induced in cells lacking factors involved in heterochromatin formation, such as RNAi (Ago1) or H3K9 HMT (Clr4) –defective cells (Supplementary Figure S2) (45,49,51). Thus, we conclude that similar to *tgp1^+^*, the *pho1^+^* gene is regulated by upstream lncRNA transcription-mediated interference in wild-type cells, not by transient heterochromatin formation. However, we can confirm that H3K9 methylation is indeed detected above background at the *pho1^+^* promoter in cells lacking the Rrp6 subunit of the exosome complex (Supplementary Figure S2). These results suggest that heterochromatin accumulation might be a secondary mechanism contributing to *pho1^+^* regulation in conditions that reduce or impair exosome function.

**Figure 7.**
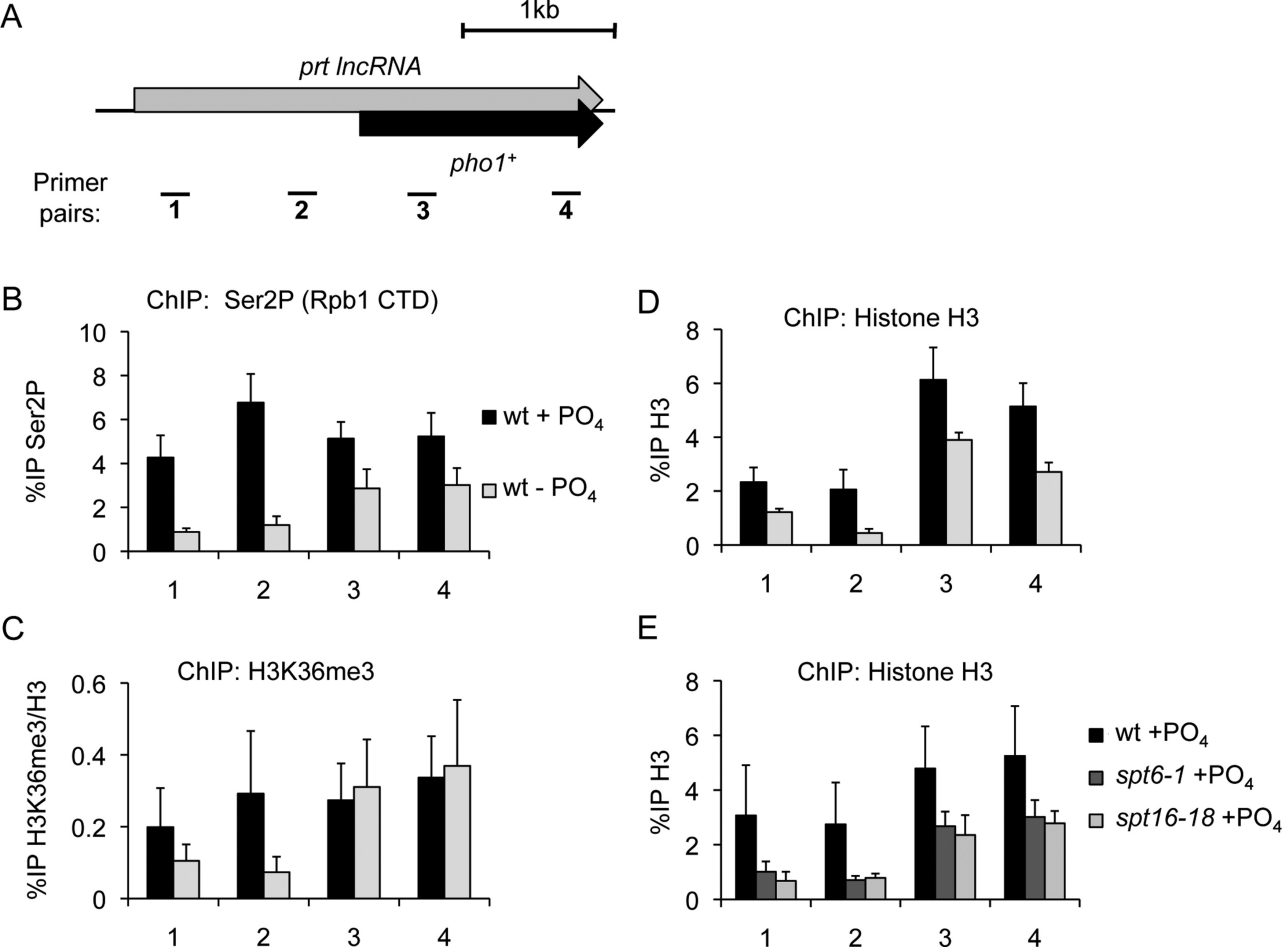
lncRNA transcription regulates *pho1^+^* by transcriptional interference. (**A**) Schematic depiction of the *pho1^+^* locus, including the lncRNA *prt* gene that occupies the *pho1^+^* promoter and overlaps the gene body downstream. Primer pairs spaced over the *pho1^+^* locus are displayed below. (**B**) ChIP-qPCR for Ser2P, the elongating form of RNAPII, over the *pho1+* locus in response to changes in phosphate availability. (**C**) Nucleosome density over the *pho1+* locus was measured by histone H3 ChIP-qPCR experiments in wild-type cells grown in the presence or absence of phosphate. (**D**) ChIP-qPCR for H3K36me3 levels over the *pho1+* locus in phosphate-replete and phosphate-starved wild-type cells. (**E**) Histone density was measured by histone H3 ChIP-qPCR experiments in wild-type cells, Stp6 mutant cells (*spt6-1*), and FACT mutant cells (*spt16-18*) grown in the presence of phosphate and at the restrictive temperature of 36°C. Error bars represent standard deviation resulting from at least three independent replicates.

**Figure 8.**
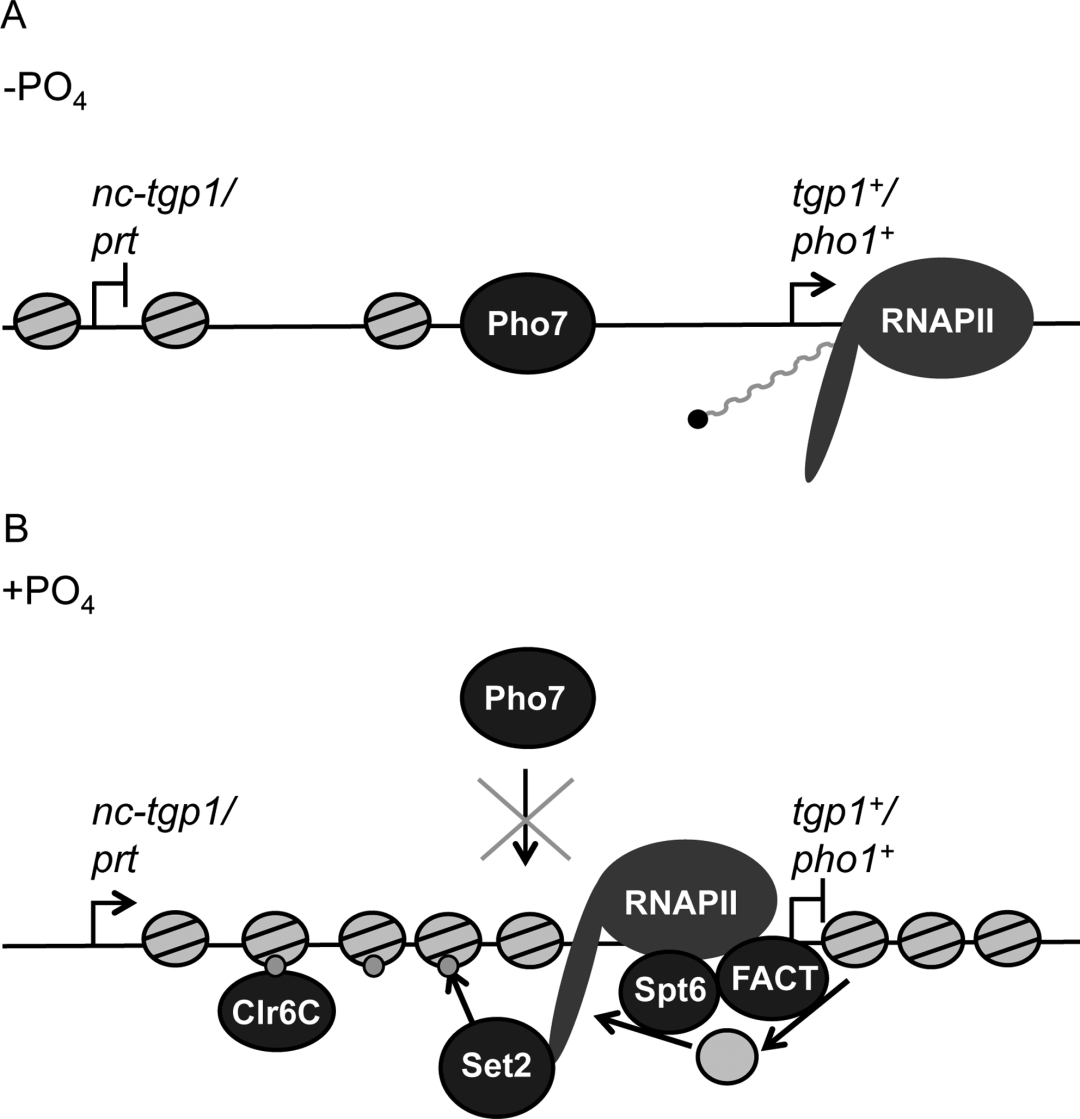
Model for transcriptional interference at *tgp1^+^* and *pho1^+^* in *S. pombe*. (**A**) Reduced upstream lncRNA transcription corresponds with decreased nucleosome density over the *tgp1^+^* and *pho1^+^* promoters to permit expression of these phosphate response genes following phosphate-starvation. (**B**) In phosphate-replete conditions, lncRNA transcription upstream of *tgp1^+^* and *pho1^+^* results in transcription-coupled deposition of Set2-dependent H3K36me3 and recruitment of the Clr6 HDAC (Clr6CII). In addition, histone chaperones Spt6 and FACT travel with RNAPII and reorganize nucleosomes during lncRNA transcription in order to limit Pho7 transcription factor binding and suppress downstream gene expression.

ChIP analyses further support *pho1^+^* regulation by transcriptional interference since the levels of Rpb1-Ser2P, histone H3, and H3K36me3 were all substantially reduced over the *pho1^+^* promoter in wild-type cells following phosphate starvation (Figure 7B–D). Moreover, we observed decreased histone H3 levels over the *pho1^+^* locus in phosphate-replete cells lacking either Spt6 or FACT activity (Figure 7E). These results suggest that transcription initiating upstream of *pho1^+^* mediates increased nucleosome density and H3K36 methylation over the *pho1^+^* promoter when *S. pombe* cells are grown in the presence of ample phosphate. We conclude that similar transcription interference mechanisms are employed to regulate the expression of both the *tgp1^+^* and *pho1^+^*genes in response to phosphate availability (See model in Figure 8).

### The Pho7 activator is required for the full induction of *tgp1*^+^ and *pho1*^+^

Finally, we sought to determine the dependency of *tgp1*^+^ and *pho1*^+^ activation on the transcription factor Pho7 in cells lacking elongation factors required for their efficient gene repression by transcriptional interference. While *tgp1*^+^ and *pho1*^+^ are partially activated in *set2Δ* or *spt6-1* cells (Supplementary Figure S3), the removal of Pho7 from these cells reduced the level of *tgp1*^+^ induction and completely prevented *pho1*^+^ expression from exceeding wild-type repressed levels (Supplementary Figure S3). This difference in dependence of *tgp1*^+^ and *pho1*^+^ activation on Pho7 is expected as partial *tgp1^+^* induction has previously been observed following phosphate-starvation in *pho7Δ* cells, whereas *pho1^+^* expression is fully dependent on Pho7 (43) (Supplementary Figure S3). We therefore conclude that loss of these transcription elongation factors is not sufficient to completely circumvent the requirement for the Pho7 activator. Since genetic manipulations that prevent *nc-tgp1* transcription brings about reduced local nucleosome density and leads to increased Pho7 binding at the *tgp1^+^* promoter in phosphate-rich conditions (34), our results are most consistent with a model whereby changes to chromatin mediated by lncRNA transcription prevent stable Pho7 binding (Figure 8).

## DISCUSSION

The orientation of closely arranged protein-coding genes in dense yeast genomes means that most acts of transcription impinge on the activity of nearby genes. For instance, non-coding antisense transcripts emanating from bidirectional gene promoters can interfere with the activation of adjacent genes and insulate against interfering acts of transcription (55,56). While networks of closely arranged genes are less prevalent in higher eukaryotes, intergenic regions are still pervasively transcribed (57). In fact, pervasive eukaryotic transcription implies that transcriptional interference mediated by intergenic lncRNA transcription might be a much more general feature of gene regulation in higher eukaryotes than is currently appreciated. Individual examples of this phenomenon have been observed in diverse biological systems ranging from free-living unicellular yeasts to multicellular organisms, including mammals (33,34,36–38,58–60). In addition, the genetic disease alpha thalassemia is caused by an intergenic single nucleotide polymorphism that creates a new promoter and initiates novel transcription that interferes with the expression of the alpha globin gene downstream (61). Transcriptional interference also plays a role in controlling human immunodeficiency virus-1 (HIV-1) latency (62,63). More recently, cells infected with herpes simplex virus-1 (HSV-1) and renal cancer cells have been observed to display defects in transcription termination that result in genome-wide read-through transcription (64,65), which is capable of repressing adjacent genes by transcriptional interference (66). Collectively, these findings suggest that transcriptional interference is a widely conserved mechanism for modulating gene expression and plays an important role in human health and disease. Nevertheless, it is remains unclear how widespread concerted gene regulation by this specific mechanism actually is. In order to determine the prevalence of transcriptional interference in eukaryotic genomes, greater mechanistic insight is required in order to pinpoint the features associated with this elusive process.

Research from diverse organisms suggests that transcription elongation is itself too rapid to mediate strong repression of downstream genes (67). In bacteria, interference between two transcription units is achieved primarily by upstream transcription pausing, which occludes underlying promoter sequences (68). While most transcription factors in prokaryotes recognize long sequence motifs (up to 30 nt or longer) (69), eukaryotic transcription factors generally recognize much shorter motifs (70). Such short regulatory elements could more easily escape occlusion by stalled RNAPII alone, which might explain why examples of transcriptional interference in eukaryotes frequently implicate transcription-associated changes in chromatin. For example, repression of the *S. cerevisiae SER3* gene by transcriptional interference requires histone chaperones, such as Spt6 and FACT, to bring about increased nucleosome density over the *SER3* promoter and prevent transcription factor binding (71). Here we show that the conserved counterparts of Spt6 and FACT are required to mediate transcriptional interference of two genes in *S. pombe* (*tgp1^+^* and *pho1^+^*) (Figures 5 and 6). This requirement for Spt6 and FACT is consistent with earlier observations demonstrating that both factors disassemble/reassemble nucleosomes in a manner that reduces transcription from cryptic promoters within active transcription units (50). Moreover, Spt6 facilitates Set2-dependent H3K36 methylation (46,72), which is required for optimal repression of *tgp1^+^* and *pho1^+^* by transcriptional interference (Figures 3 and 6). The requirement for Set2 to mediate transcriptional interference in *S. cerevisiae* is less clear. While repression of the *S. cerevisiae IME1* gene requires interfering transcription-coupled H3K36me3 deposition by Set2 (60), suppression of the *SER3* gene does not require Set2 activity (71). To explain this discrepancy, it has been proposed that the lncRNA upstream of *SER3* is too short (only ∼500 nt) to allow the transcriptional elongation phase of RNAPII to deposit sufficient H3K36me3 over the *SER3* promoter (60). This explanation is supported by the fact that Set2/Rpd3S predominantly limits intragenic transcription initiation on longer genes (73). Our data strengthen the concept that Set2 is required for transcriptional interference involving longer lncRNAs since the transcripts mediating Set2-dependent *tgp1^+^* and *pho1^+^* repression are each initiated ∼2 kb upstream of their respective target gene (34,51). It is also possible that the act of RNAPII progression through a promoter may contribute to the displacement and/or limit transcription factor binding, however, our findings in *S. pombe* along with those in the evolutionarily distant *S. cerevisiae* demonstrate that transcription elongation-associated chromatin changes are pivotal for mediating transcriptional interference in eukaryotes.

Since numerous elongation factors that are known to prevent intragenic transcription initiation also contribute to the effectiveness of gene silencing by transcriptional interference, we suggest that gene promoters occluded by upstream initiating transcripts are in essence themselves ‘cryptic promoters’ residing within the interfering transcription unit. Importantly, elongation factors are widely conserved in eukaryotes. A prediction therefore is that the basic mechanistic features of transcriptional interference revealed in both *S. pombe* and *S. cerevisiae*, two highly divergent yeast species separated by hundreds of millions of years of evolution, will also be conserved in metazoans. Indeed, Spt6 and FACT have already been implicated in transcriptional interference of the *Ubx* gene in *Drosophila* (38), while H3K36 methylation appears to contribute to transcriptional interference in mammals (33,64). Extensive analyses are required to identify additional components of the regulatory circuit that promotes transcriptional interference. Such information will provide a much more comprehensive understanding of the underlying mechanism, in addition to explaining mechanistic variability (such as the involvement or not of Set2). Furthermore, conserved features of transcriptional interference might provide useful hallmarks for assessing how widespread this regulatory mechanism truly is in various eukaryotic genomes. Maps of the genomic locations of specific transcription-coupled histone modifications along with factors known to be involved in mediating transcriptional interference (e.g. Set2, Spt6, FACT) should help to uncover additional examples of genes regulated by this mechanism. Complementary methods, such as native elongating transcript sequencing (NET-seq), which allows nascent transcript locations to be determined (74), should also facilitate the identification of novel interfering upstream transcripts. Together, we expect that such approaches should provide information regarding the potential scale and prevalence of transcriptional interference in different genomes. Finally, examples of transcriptional interference associated with diverse human diseases (61–65) imply that a more comprehensive understanding of the underlying mechanism of this form of gene regulation should better inform the molecular basis of pathologies that result from alterations in transcription dynamics.

## Supplementary Material

SUPPLEMENTARY DATA
