# Supplementary material for: Transcription-coupled changes to chromatin underpin gene silencing by transcriptional interference
Source: Nucleic Acids Res. 2016 Sep 8;44(22):10619–30. doi: 10.1093/nar/gkw801 (PMC5159543; doi:10.1093/nar/gkw801)
Supplement: SUPPLEMENTARY DATA [file supp_44_22_10619__index.html]

Transcription-coupled changes to chromatin underpin gene silencing by transcriptional interference — Transcription-coupled changes to chromatin underpin gene silencing by transcriptional interference — SUPPLEMENTARY DATA 

# Transcription-coupled changes to chromatin underpin gene silencing by transcriptional interference

## SUPPLEMENTARY DATA

- SUPPLEMENTARY DATA
