# Supplementary material for: Transcription-coupled changes to chromatin underpin gene silencing by transcriptional interference
Source: Nucleic Acids Res. 2016 Sep 8;44(22):10619–30. doi: 10.1093/nar/gkw801 (PMC5159543; doi:10.1093/nar/gkw801)
Supplement: SUPPLEMENTARY DATA [file supp_gkw801_nar-01308-x-2016-File002.pdf]

SUPPLEMENTARY MATERIAL

Title: **Transcription-coupled changes to chromatin underpin gene silencing by transcriptional interference**

Authors: Ryan Ard and Robin Allshire

Supplementary Figures 1 – 3 (pages 2 – 4)

Supplementary Tables 1 and 2 (pages 5 and 6)

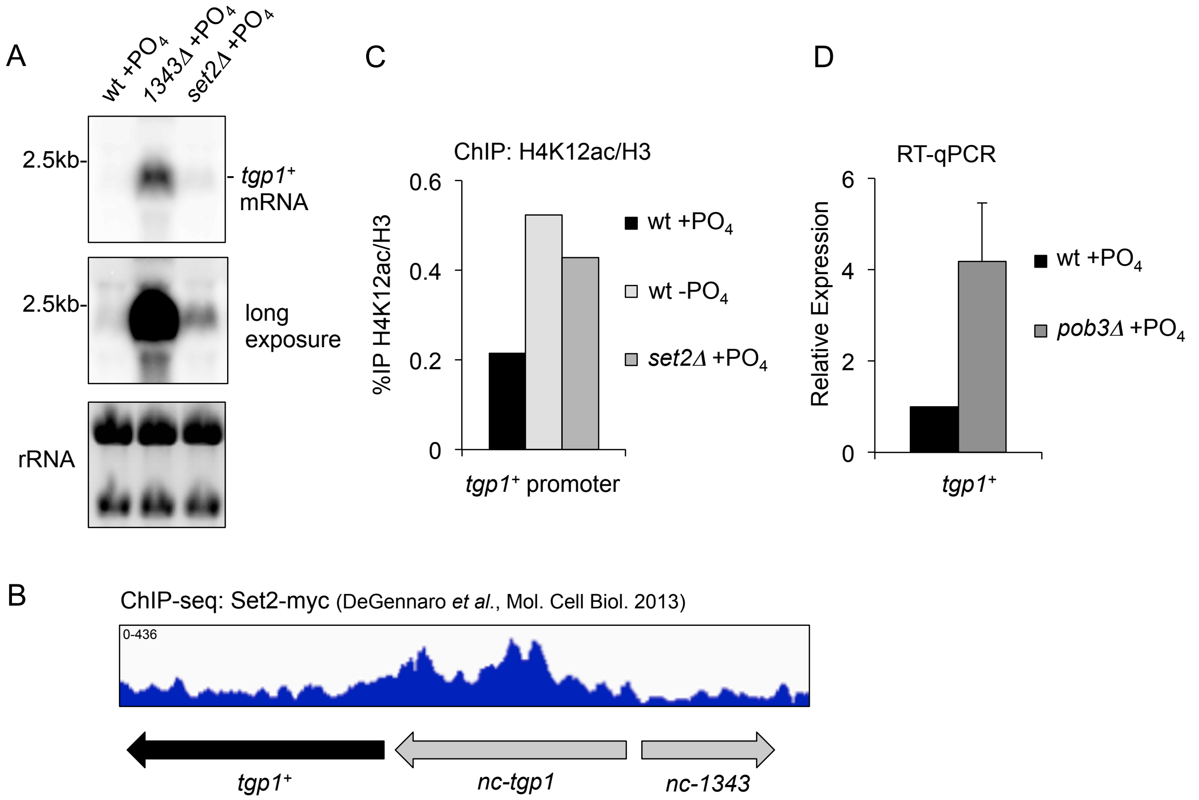

Supplemental Figure 1. Transcription elongation factors contribute to *tgp1$^+$* regulation. (A) Northern analysis of *tgp1$^+$* mRNA levels in wild-type cells, *1343Δ* cells with constitutive *tgp1$^+$* expression (i.e., cells lacking the promoter that drives repressive *nc-tgp1* transcription), and in *set2Δ* cells. (B) Set2 enrichment over the *tgp1$^+$* locus as determined by previous ChIP-seq analyses [46]. (C) The levels of acetyl-histones (H4K12ac) at the *tgp1$^+$* promoter primer pair 3 (see Figure 1) were measure by ChIP-qPCR in wild-type cells grown in the presence or absence of phosphate and *set2Δ* cells grown in the presence of phosphate. (D) RT-qPCR analysis comparing *tgp1$^+$* mRNA levels in wild-type cells with levels detected in cells lacking the FACT complex subunit Pob3 (*pob3Δ*). Error bars represent standard deviation resulting from at least three independent replicates.

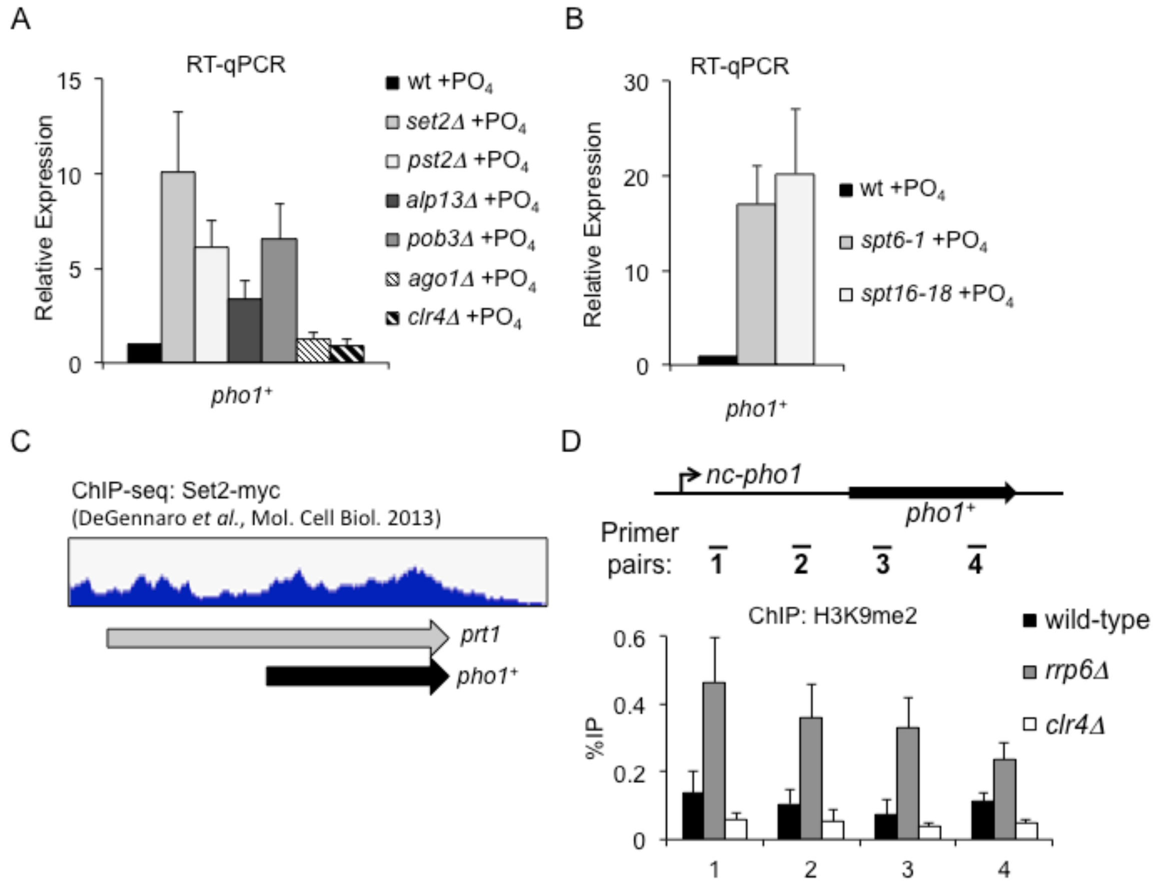

Supplemental Figure 2. Loss of elongation factors induces *pho1*$^{+}$ expression. (A) RT-qPCR analysis of *pho1*$^{+}$ mRNA levels in phosphate-replete wild-type cells, *set2Δ*, *pst2Δ*, *alp13Δ*, *pob3Δ*, and cells lacking either RNAi component Ago1 (*ago1Δ*) or H3K9 HMT Clr4 (*clr4Δ*) (B) RT-qPCR analysis comparing *pho1*$^{+}$ mRNA levels in phosphate-replete wild-type cells, *spt6-1* cells, and *spt16-18* cells shifted to the restrictive temperature of 36°C for 1 hr. (C) Set2 enrichment over the *pho1*$^{+}$ locus as determined by previous ChIP-seq analyses [46]. (D) H3K9me2 ChIP-qPCR experiments performed in wild-type and *rrp6Δ* cells. Cells lacking the H3K9 HMT Clr4 (*clr4Δ*) cells were used as a negative control to measure the background level of noise signal in the experiment. Error bars represent standard deviation resulting from at least three independent replicates.

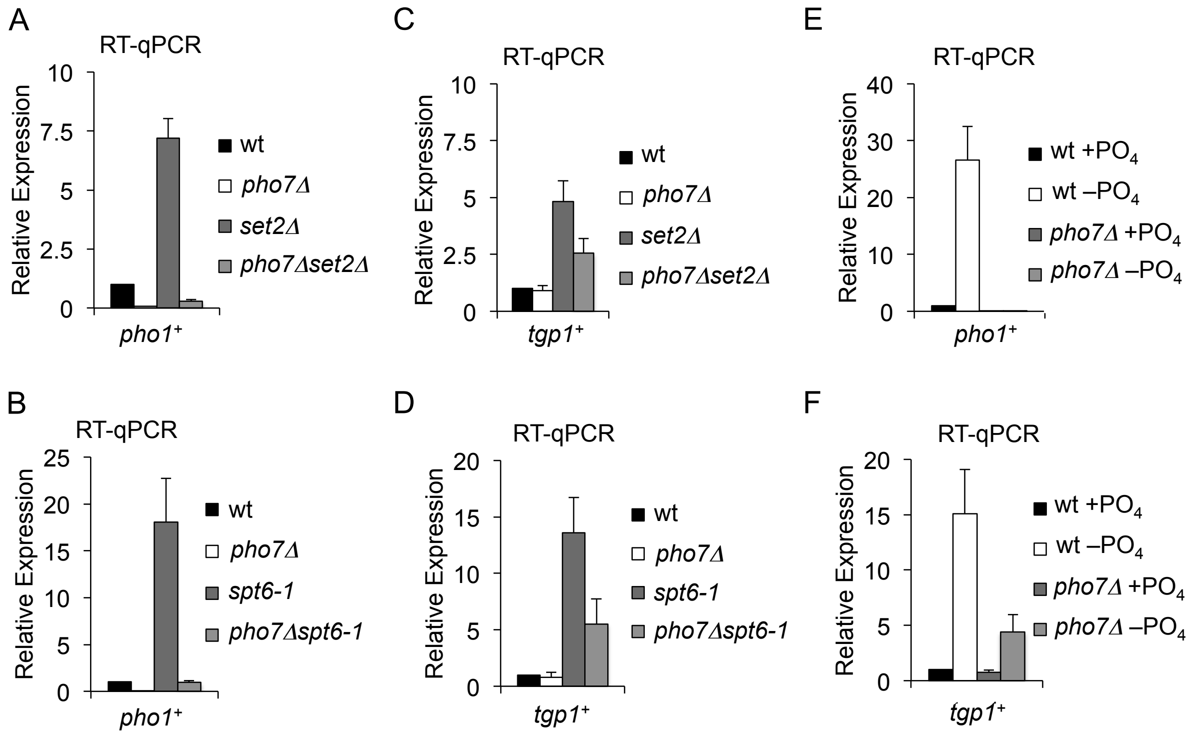

Supplemental Figure 3. Dependency of transcription factor Pho7 on *tgp1$^+$* and *pho1$^+$* activation. (A) RT-qPCR analysis of *pho1$^+$* mRNA levels in phosphate-replete wild-type cells, *pho7Δ*, *set2Δ*, and the *pho7Δset2Δ* double mutant. (B) RT-qPCR analysis of *pho1$^+$* mRNA levels in phosphate-replete wild-type cells, *pho7Δ*, *spt6-1*, and the *pho7Δspt6-1* double mutant shifted to the restrictive temperature of 36°C for 1 hr. (C) RT-qPCR analysis of *tgp1$^+$* mRNA levels in phosphate-replete wild-type cells, *pho7Δ*, *set2Δ*, and the *pho7Δset2Δ* double mutant. (D) RT-qPCR analysis of *tgp1$^+$* mRNA levels in phosphate-replete wild-type cells, *pho7Δ*, *spt6-1*, and the *pho7Δspt6-1* double mutant shifted to the restrictive temperature of 36°C for 1 hr. (E) RT-qPCR analysis of *pho1$^+$* mRNA levels in wild-type cells and *pho7Δ* cells grown in the presence or absence of phosphate. (F) RT-qPCR analysis of *tgp1$^+$* mRNA levels in wild-type cells and *pho7Δ* cells grown in the presence or absence of phosphate. Error bars represent standard deviation resulting from at least three independent replicates.

Supplementary Table 1. List of *S. pombe* strains used in this study.

Name	ID #	Genotype	Source
wild-type	1645	*h+ ade6-210 arg3-D4 his3-D1 leu1-32 ura4-D18*	Lab stock
wild-type	1646	*h- ade6-210 arg3-D4 his3-D1 leu1-32 ura4-D18*	Lab stock
1343Δ	A9032	*h+ SPNCRNA.1343Δ ade6-210 arg3-D4 his3-D1 leu1-32 ura4-18*	Lab stock
tgp1Δ	A9352	*h+ 1343Δ tgp1Δ::ura4^{+} ade6-210 arg3-D4 his3-D1 leu1-32 ura4-D18*	Lab stock
nmt1-nc-tgp1	B0200	*h- nc-tgp1-promoter:nmt1-NAT ade6-210 arg3-D4 his3-D1 leu1-32 ura4-D18*	Lab stock
set2Δ	8666	*h- set2::Kan leu1-32 ade6-210 uraD18*	Lab stock
tgp1Δset2Δ	B1199	*h? tgp1::ura4 set2::Kan leu1-32 ade6-210 uraD18*	This study
pst2Δ	A2858	*h+ pst2::KAN ade6-210 leu1-32 uraD18 his3D1 arg3-D4*	Lab stock
spt6-1	A6793	*h- spt6-1-NAT ade6-210 leu1-32 uraD18*	Winston, F.
spt16-18	A5259	*h- spt16-18-KAN leu1-32*	Lab stock
alp13Δ	3802	*h+ alp13::ura4 ade6-210 ura4D18 leu1-32*	Toda, T.
pob3Δ	A6353	*h? pob3::NAT 5myc-cnp1*	Lab stock
ago1Δ	8061	*h+ ago1Δ::ura4 otr1R(SphI):ade6+ ura4-D18 leu1-32 ade6-M210*	Lab stock
clr4Δ	8435	*h- clr4Δ::ura4 his7-366 ade6-210/216 leu1-32 ura4-D18*	Lab stock
rrp6Δ	7865	*h+ rrp6Δ::KAN ade6-210 ura4-D18 leu1-32*	Lab stock
pho7Δ	B1447	*h- pho7Δ::KAN*	Wykoff, D.
pho7Δset2Δ	B1454	*h? pho7Δ::KAN set2Δ::KAN*	This study
pho7Δspt6-1	B1455	*h? pho7Δ::KAN spt6-1-NAT*	This study

Supplementary Table 2. List of oligonucleotides used in this study.

Name	Sequence
qAct1_F	GGTTTCGCTGGAGATGATG
qAct1_R	ATACCACGCTTGCTTTGAG
qTgp1_1F (PP: 1*)	TCGGTTGGAATGTTCTAATCAATAC
qTgp1_1R (PP: 1*)	AGACCGGTGATCAAACAATATTTAG
qTgp1_2F (PP: 2*)	TGAAGTAGTTAGACAGGTTAGCGA
qTgp1_2R (PP: 2*)	CTTGTCGTCCAACTTCTCTTCATC
qnctgp1c_F (PP: 3)	GGCAGTAAATCTATCTGTAGCGAGT
qnctgp1c_R (PP: 3)	TACACGGTAAATGTCAAGTCTGCTA
qnctgp1b_F (PP: 4*)	CTGACAAACCAATTATCCCTACACG
qnctgp1b_R (PP: 4*)	GTATTACGATTTGGCAACCTCATCC
qnctgp1a_F (PP: 5*)	TTAAATGCTGCACTCACATACTGAC
qnctgp1a_R (PP: 5*)	ACTCTCCCTTGGGTTCATTTGATTA
qPho1_1F	CTTTGGACCCTCTAATACATCCGAT
qPho1_1R	AAGAGTGTCAAAGTTCTGGATACCA
qPho1_2F	AAGATTCTAAGTACTATGTCCGCCA
qPho1_2R	ATCGGATGTATTAGAGGGTCCAAAG
qncPho1_1F	ATGATGTTTGAGATTTACGGGAAGT
qncPho1_1R	TTCTGTAAATGTGTCCCGAACCAAA
qncPho1_2F	ATGATGTTTGAGATTTACGGGAAGT
qncPho1_2R	TTCTGTAAATGTGTCCCGAACCAAA
nbTgp1_F	ATGGTTACTGCTCCAATTCAATCGG
nbTgp1_R	TAATACGACTCACTATAGGGAGAAATCAATGGCACCGTCCGTAAC
nbPho1_F	CGTTGTAGATTCTGCTCTTTGGT
ncPho1_R	TAATACGACTCACTATAGGGAGAAGGCAATGGATTCTCAGGAGTAT
